# Supplementary material for: Splice-Junction-Based Mapping of Alternative Isoforms in the Human Proteome
Source: Cell Rep. Author manuscript; Available in PMC 2020 Jan 15. (PMC6961840; doi:10.1016/j.celrep.2019.11.026)
Supplement: 2 [file NIHMS1546469-supplement-2.zip › DF1/PXD010154/Heart/Heart_24_RYR2_VLDIANVLFHLEQVEHPQR.pdf]

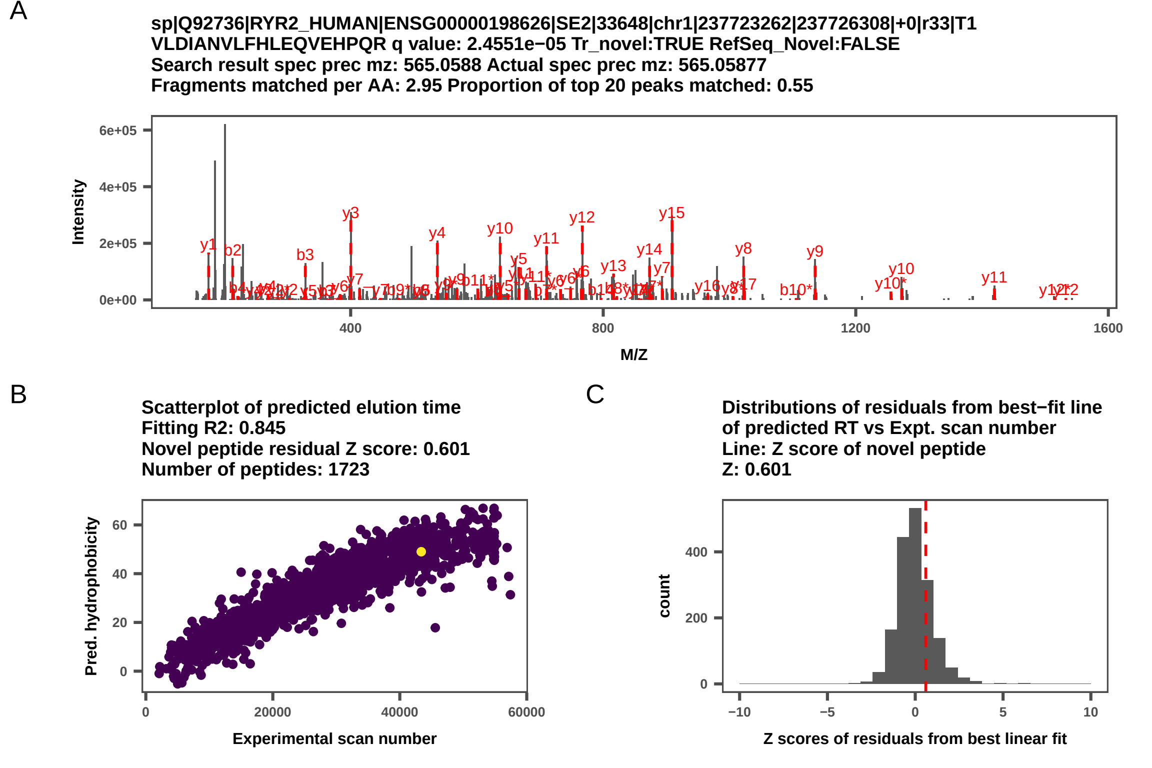
A
sp|Q92736|RYR2_HUMAN|ENSG00000198626|SE2|33648|chr1|237723262|237726308|+0|r33|T1
VLDIANVLFHLEQVEHPQR q value: 2.4551e−05 Tr_novel:TRUE RefSeq_Novel:FALSE
Search result spec prec mz: 565.0588 Actual spec prec mz: 565.05877
Fragments matched per AA: 2.95 Proportion of top 20 peaks matched: 0.55
Intensity
6e+05
4e+05
2e+05
0e+00
y1
b2
b3
y3
y4
y10
y11
y12
y15
y14
y13
y8
y9
y10
y11
y5
y7
b10*
y16
M/Z
400
800
1200
1600
B
Scatterplot of predicted elution time
Fitting R2: 0.845
Novel peptide residual Z score: 0.601
Number of peptides: 1723
Pred. hydrophobicity
60
40
20
0
0
20000
40000
60000
Experimental scan number
C
Distributions of residuals from best−fit line
of predicted RT vs Expt. scan number
Line: Z score of novel peptide
Z: 0.601
count
400
200
0
−10
−5
0
5
10
Z scores of residuals from best linear fit
